# Nonlinear field-control of terahertz waves in random media for spatiotemporal focusing

**DOI:** 10.12688/openreseurope.14508.1

**Published:** 2022-03-08

**Authors:** Vittorio Cecconi, Vivek Kumar, Alessia Pasquazi, Juan Sebastian Totero Gongora, Marco Peccianti

**Affiliations:** 1 Emergent Photonics (EPic) Lab, Department of Physics and Astronomy, University of Sussex, Brighton, BN19QH, UK

**Keywords:** Scattering, terahertz, time-domain spectroscopy, random medium, spatiotemporal focusing, superfocusing, genetic algorithm

## Abstract

Controlling the transmission of broadband optical pulses in scattering media is a critical open challenge in photonics. To date, wavefront shaping techniques at optical frequencies have been successfully applied to control the spatial properties of multiple-scattered light. However, a fundamental restriction in achieving an equivalent degree of control over the temporal properties of a broadband pulse is the limited availability of experimental techniques to detect the coherent properties (i.e., the spectral amplitude and absolute phase) of the transmitted field. Terahertz experimental frameworks, on the contrary, enable measuring the field dynamics of broadband pulses at ultrafast (sub-cycle) time scales directly. In this work, we provide a theoretical/numerical demonstration that, within this context, complex scattering can be used to achieve spatio-temporal control of instantaneous fields and manipulate the temporal properties of single-cycle pulses by solely acting on spatial degrees of freedom of the illuminating field. As direct application scenarios, we demonstrate spatio-temporal focusing, chirp compensation, and control of the carrier-envelope-offset of a transform-limited THz pulse.

## Plain language summary

Multiple scattering of light is a common phenomenon in everyday life. The opaqueness of fog, milk, and clouds are excellent examples of how light is scrambled when travelling through materials composed of thousands of particles scattering light in all directions. While scattering is generally perceived as unwanted, researchers have shown that complex but inexpensive substances like white paint or frosted glass can be adapted to behave as expensive optical devices, such as high-resolution lenses or optical computing devices. This surprising result is achieved by using particular projectors on the incident light to impress specific patterns yielding a desired shape at the output. While manipulating the spatial profile of light is now well-understood, controlling how scattering affects ultra-short light pulses remains exceptionally challenging, primarily due to the difficulty in measuring the effects of scattering at the ultrafast timescales of the pulse (quadrillionths of a second).

In this theoretical work, we propose a new way to tackle this challenge by leveraging the unique properties of terahertz light waves. Terahertz light lies between microwaves and infrared in the electromagnetic spectrum and is highly sought in research and industry. It can easily reveal the material composition of an object and penetrate common materials like paper and plastic as X-rays do, but without being harmful. More importantly, we can experimentally detect the individual oscillations in a terahertz pulse and extract an unprecedented understanding of how scattering affects it.

We show that this added knowledge gives us the ability to finely manipulate the properties of the pulse, compensate the pulse-broadening effects of scattering, and “engineer” specific pulse shapes, a highly-sought ability in photonics. In the long term, this ability could be applied to reveal the internal composition of complex samples, such as biological tissue, or extract images of samples placed behind (or within) scattering materials.

## Introduction

Multiple scattering is generally perceived as detrimental in photonics, as it is commonly associated with unpredictability and loss of information. For instance, in microscopy or astronomy, scattering is widely known to severely affect the resolution and fidelity of an imaging system ^1, 2^. As a result, a large body of research has historically focused on compensating or straight-out eliminating scattering effects ^3– 5^. In a stark paradigm shift, researchers have recently demonstrated that disordered media can become essential ingredients in developing photonic devices with sophisticated optical performances ^6– 9^. These include, among others, scattering-assisted super-resolution imaging lenses, wavefront-shaping components, or optical neuro-computing devices ^10– 18^. At their heart, these demonstrations rely on wavefront shaping techniques, i.e., on the identification of an optimal incident field distribution, or pattern, yielding a desired intensity distribution at the output of a scattering medium ^19, 20^. While the ability to control monochromatic beams' spatial properties has matured recently, manipulating the temporal and spectral properties of the scattered field remains challenging, particularly when considering broadband ultrafast illumination ^21– 25^. Multiple scattering is an inherently dispersive phenomenon that naturally leads to the broadening of optical pulses ^26, 27^. Recent works have shown that the intrinsic coupling between spatial and temporal dimensions in scattering media can be leveraged to manipulate the temporal properties of pulses by controlling the spatial features of the illumination ^28, 29^. As a result, spatial wavefront shaping can be applied to achieve spatiotemporal focusing, corresponding to a simultaneous focusing in space and pulse re-compression in time, either through iterative approaches or by measuring the frequency-dependent transmission matrix of the sample ^21, 30– 32^. These approaches have shown how to control the envelope of the transmitted waveform successfully (e.g., to adjust the centre of the transmitted pulse ^32– 35^), but they remain unsuitable for manipulating the carrier-wave properties (e.g., the carrier-envelope offset of the transmitted pulse). Further advances in this area are fundamentally hindered by the inability to directly measure the full-wave properties of the scattered field, most notably the absolute spectral phase ^23, 35^. An interesting question is whether a direct measurement of the electric field oscillations could enable controlling the coherent features of the transmitted pulse and devising advanced forms of waveform synthesis and spectral shaping currently out-of-reach at optical frequencies. In this context, field-sensitive detection is well-established in terahertz (THz) photonics, where time-domain spectroscopy (TDS) grants access to the time-resolved detection of the electric field of single-cycle THz pulses ^36^. Leveraging this ability, THz-TDS has been applied to study the broadband properties of scattering samples, with a particular emphasis on the role of multiple scattering in biomedical applications ^26, 27, 37– 40^ and the effects of resonant excitation of the scatterers composing the medium ^41– 45^. However, the implementation of a complete, field-based wavefront control methodology to manipulate broadband pulses is essentially unexplored in the THz frequency band. In this work, we provide a first theoretical exploration of the potential advantages offered by time-resolved, field-sensitive detection in manipulating broadband THz pulses using scattering media. We combine the nonlinear generation of THz patterns from structured optical beams with an evolutionary optimisation feedback-loop targeting the coherent, full-field properties of the transmitted field, showcasing the ability to achieve spatiotemporal focus and control the absolute phase of the transmitted pulse. Quite interestingly, access to the absolute spectral phase of the pulse enables the definition of effective spectral shaping strategies, such as the ability to compress an incident chirped-pulse or control the carrier-envelop offset (CEO) of an incident pulse, an extremely challenging task at optical frequencies.

## Physical framework and methodology

### Problem definition: full-wave control of THz pulses in complex media

We model the linear transmission properties of the scattering medium through a dispersive transmission operator *TM* ( *x, y, ω*) ^33, 46^. For the sake of simplicity, we focus on a scalar description of the scatterer, but our approach can be easily extended to a full-vector formulation ^47^.

In the presence of an input field distribution *E* ^-^ ( *x, y, ω*), the field transmitted through the scatterer *E* ^+^ ( *x, y, ω*) is expressed through a (spatial) convolution relation:


$$E^{+}(x,y,\omega )=\int dx^{\prime }dy^{\prime }TM(x-x^{\prime },y-y^{\prime },\omega )E^{-}(x^{\prime },y^{\prime },\omega ).\;(1)$$

Following Ref. 48, we discretize the output and input planes in *M* and *N* two-dimensional, square pixels, respectively. In such a formulation, the spectral components of the fields are re-defined as column arrays, and Equation (1) is rewritten in terms of a frequency-dependent transfer matrix *T _mn_* ( *ω*) as follows: 


$$\;E_{m}^{+}(\omega )=\sum_{n=k}^{N}TM_{mn}(\omega )\cdot E_{n}^{-}(\omega ),\;(2)$$

where $E_{m}^{+}$ $E_{n}^{-}$ represents the field in the *m*-th ( *n*-th) pixel of the output (input) field distributions. Following standard approaches, we express the transmission matrix as a complex-valued, random Gaussian matrix: 


$$\;TM_{mn}(\omega )=\exp[i\phi_{mn}(\omega )]/N,\;(3)$$

where *ϕ _mn_* ( *ω*) is a random phase distribution uncorrelated in space and gaussian-correlated along the frequency axis. The degree of spectral correlation, generally expressed in terms of the spectral correlation bandwidth Δ *v _c_*, is commonly employed to characterise the spectral response of a scattering medium, and it is generally defined as the inverse of the Thouless time ^24^. In practice, a frequency-correlated transfer matrix can be modelled numerically by applying a Gaussian spectral filter of width Δ *v _c_* to a white-noise random matrix ^49, 50^.

**Figure 1.**
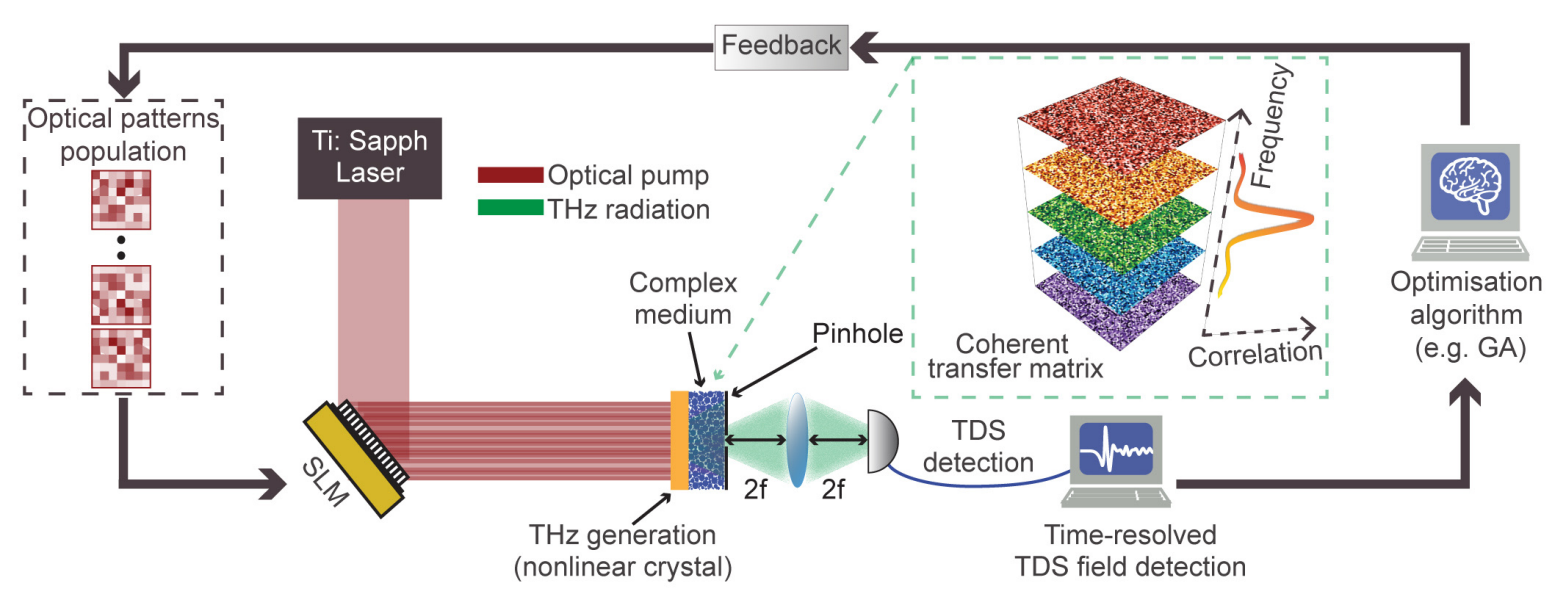
Nonlinear wavefront control of THz complex media and conceptual overview of the iterative algorithm. Illustrative imaging setup, including a nonlinear crystal (yellow) emitting structured THz waves (green) generated from optical patterns (red) through a standard spatial light modulator (SLM). The transmitted THz field is collected via time-domain spectroscopy (TDS). The generation crystal is placed close to the scattering medium. The black arrows describe the schematic loop of the iterative identification of an optimal pattern producing a pulse with the desired spatiotemporal properties via a genetic algorithm (GA) approach. In the green-dashed box, we show a conceptual picture of the hyperspectral transfer matrix and its frequency correlation.

In general terms, a wavefront control approach aims to identify the optimal incident field distribution $E_{optimal}^{-}(x,y,t)$ yielding the desired output field $E_{target}^{+}(x,y,t)$ at the output facet of the scatterer ^51^. However, when operating at THz frequencies, the ability to control the incident electric field distribution is hindered by the limited availability of spatial light modulator (SLM) devices. To overcome this limitation, we employ the nonlinear conversion of structured optical beams, an approach we have recently developed within the framework of time-resolved nonlinear ghost imaging (NGI) ^52– 54^. With this approach, any optical pattern generated through a standard SLM device can act as a direct source of broadband THz patterns. By considering a nonlinear quadratic process (e.g., in a *χ* ^(2)^ crystal such as ZnTe), the relation between incident optical intensity and generated THz field is linear, i.e., *E _THz_* ( *x, y, t*) ∝ *I _pumb_* ( *x, y, t*), enabling the precise control over the THz field profile by simply shaping the incident optical pulse. A key advantage is that the spatial resolution of the THz patterns can be pushed way below the standard THz wavelength scale, as it is only bound by the diffraction limit of the optical beam. Besides, the nonlinear conversion ensures that the spatial pattern is well-defined across the whole THz band, enabling the generation and control of single-cycle, THz structured beams. We can assume that the incident THz patterns are expressed as:


$$\;E^{-}(x,y,\omega )\;\text{α}\;A_{0}\;P(x,y)f_{THz}(\omega ),\;(4)$$

where *A*_0_ is an amplitude, *P*( *x, y*) is the (all positive) spatial profile, and *f _THz_* (ω) is the spectrum of the THz pulse, which is assumed to be the same for all input points. Figure 1 outlines a possible implementation of our wavefront-control methodology ^52– 54^. The spatial pattern, impressed by an optical SLM on the pump field, is transferred to the THz pulse through nonlinear conversion in a quadratic crystal. The patterned THz field impinges on the scatterer, that is placed in close proximity (i.e., in the near-field or direct contact) of the generating crystal. Upon propagation, an electro-optical sampler collects the transmitted field in the typical fashion of standard TDS detection ^55^. As our objective is to manipulate the full-field properties of the transmitted THz pulse in a specific pixel of the output plane, we introduce a spatial pinhole at the output facet of the scatterer, corresponding to the desired focal spot ( *x*_0_, *y*_0_). To optimise the wavefront of the transmitted pulse, the collected THz field from a predetermined ensemble of patterns is analysed and ranked through an evolutionary optimisation algorithm (e.g., a genetic algorithm (GA)) that provides the feedback required to iteratively optimise the transmitted field. This configuration enables establishing an optimisation feedback loop relying on the temporal field properties instead of average intensity, as is generally the case when operating at optical frequencies. In our configuration, the combination of near-field coupling and generation of THz patterns from structured optical fields provides a significant advantage in terms of available degrees of freedom. The THz field can be densely sampled in space, with patterns reaching sub-wavelength spatial resolution in the near-field region. This is a drastic difference from typical optical embodiments, where the spatial sampling is limited by the numerical aperture of the illumination ^56^. On the contrary, in our case, the sampling can exceed the density of the modes accessible from the scatterer input facet, and the spatial density of the transfer matrix can be as high as required to represent the scattering medium accurately ^48^.

### Numerical implementation and optimisation strategy

To demonstrate this concept, we numerically simulated the configuration in Figure 1 using MATLAB 2021b. The plotting and simulation codes are fully compatible with Octave open-source software ^57^. The simulation codes require the “statistics” Octave package, freely available through the Octave Forge repository. The plotting codes require the “signal” Octave package, freely available through the Octave Forge repository. As a THz input field, we considered a transform-limited THz pulse with a duration of 1 ps, a typical product of a bandwidth-limited optical rectification process in a nonlinear crystal. The incident pulse spectrum is centred at 1 *THz* (Δ *v* ⋅ *τ _p_* ≃ 0.44, where *τ _p_* is the full width half maximum (FWHM) pulse duration and Δ *v* the bandwidth of the power spectral distribution). In terms of spatial features, we considered grayscale spatial patterns composed of 33 × 33 square pixels of side Δ *x* = 100 *μm*. The scattered field spectral correlation bandwidth is Δ *v _c_* = 250 *GHz*, a value that is compatible with experimental literature and which places us in a non-monochromatic case ^38– 40, 58^. The optimisation is performed via a standard GA targeting different fitness functions, and corresponding to the genetic algorithm described in Ref. 59. Leveraging the ability to resolve in time the transmitted electric field, we define the fitness functions maximised by the GA in terms of the statistical moments of the electric field waveform, defined as follows:


$$\;\begin{array}{c} E_{0}=\int dt|E(t)|,\\ \mu [E(t)]=\int dt\;t\frac{\left|E(t)\right|}{E_{0}},\\ \sigma [E(t)]=\sqrt{\int dt\;{\left(t-\mu \right)}^{2}\frac{\left|E(t)\right|}{E_{0}}},\\ S[E(t)]=\frac{1}{\sigma^{3}}\int dt\;{\left(t-\mu \right)}^{3}\frac{\left|E(t)\right|}{E_{0}}, \end{array}\;(5)$$

where *μ*, *σ*, and *S* are the first, second (standard deviation) and third moments (skewness) of the probability

distribution defined by | *E*( *t*)|. Practically speaking, these quantities correspond to the centre, duration, and symmetry of the pulse waveform. We stress that all those quantities do not relate to the pulse envelope or to locally averaged intensity profiles, as in the common optical practice. Table 1 summarises the different types of fitness functions employed in our analysis.

**Table 1.** The set of cost functions used in the genetic algorithm. In all these expressions, the electric field is measured in the desired focal point, i.e. *E*_0_( *t*) ≡ *E*( *x*_0_, *y*_0_, *t*). The statistical moments of the electric field *μ*, *σ*, and *S* are defined in Equation (5). For the fitness function C, the variables *α*, *β*, *γ*, and *ξ* are weights controlling the relative importance of each component of the multi-objective fitness function. For the fitness function D, *c.c.* stands for complex conjugate.

Fitness function	Definition	Type of Optimisation
A	$\frac{\max(E_{0}(t))}{\sigma [E_{0}(t)]}$	Spatiotemporal focusing
B	$-\frac{\min(E_{0}(t))}{\sigma [E_{0}(t)]}$	Spatiotemporal focusing and phase inversion
C	$\text{α}\;\max(E_{0}(t))-\beta |\mu [E_{0}(t)]-t_{0}|-\gamma |\sigma [E_{0}(t)]-\sigma_{0}|-\zeta |S[E_{0}(t)]-S_{0}|$	Achieving a desired time delay t _0_, temporal deviation σ _0_ and skeweness S _0_
D	$\frac{\int d\omega E_{0}(\omega )E_{target}^{*}(\omega )+c.c.}{\sigma [E_{0}(t)]}$	Phase-sensitive spectral shaping, where the aim is to obtain a measured field *E*_0_( *ω*) as close as possible to a target field *E _target_* ( *ω*)

## Results

### Spatiotemporal focusing and temporal shift of recompressed pulses.

**Figure 2.**
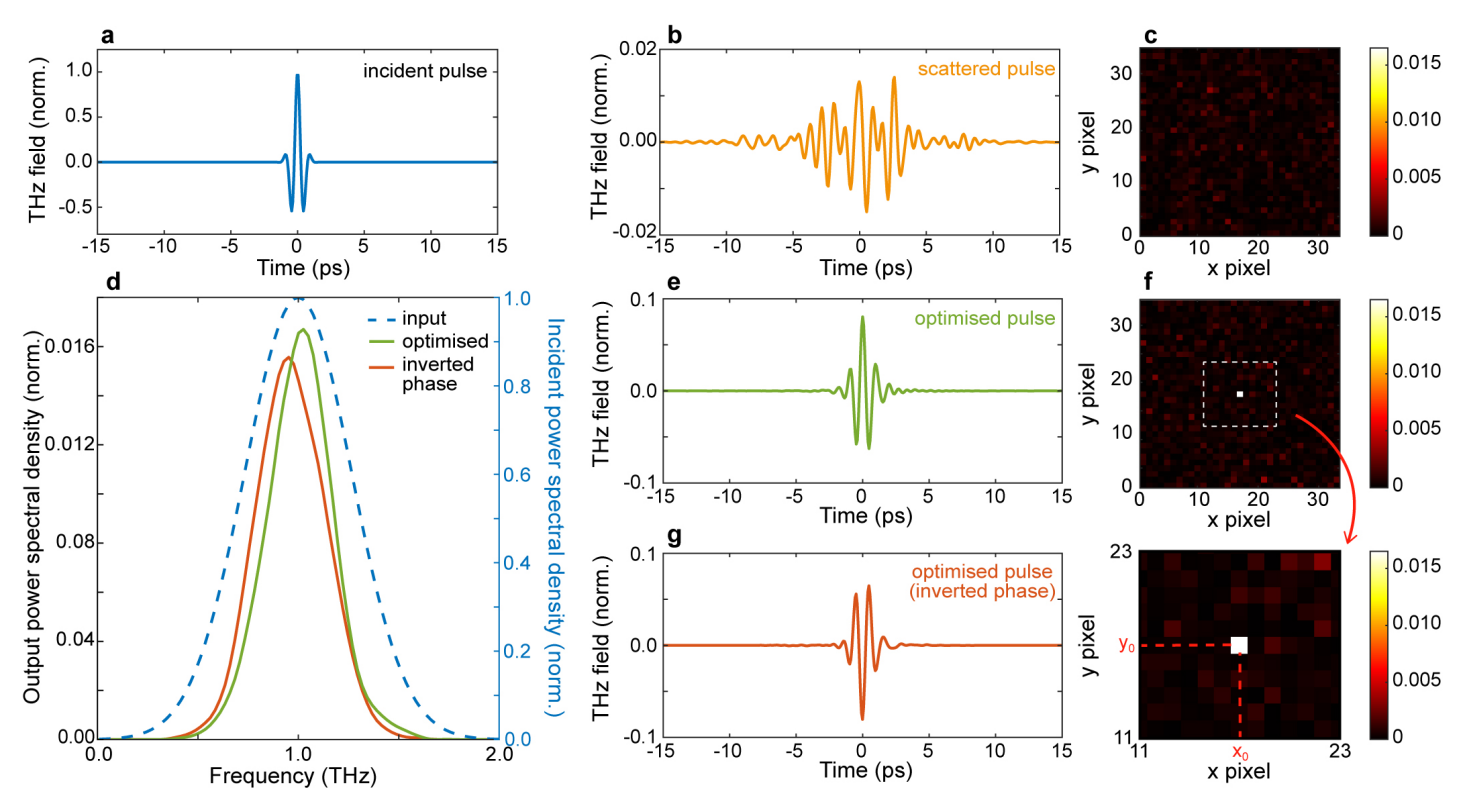
Spatiotemporal refocusing and pulse flip. **a.** Input temporal profile. **b.** Temporal profile of a scattered pulse at the output of the scattering medium; the pulse is normalised with respect to the incident pulse. **c.** Non-optimised spatial intensity distribution at 1 *THz*. **d.** Power spectral density (PSD) spectrum of the optimal terahertz pulse (green line), optimised flipped pulse (orange line) and incident pulse (blue dashed line, right y-axis). **e.** Temporal profile of an optimised pulse. **f.** Optimised spatial intensity distribution at 1 *THz*. **g.** Temporal profile of an optimised pulse with an inverted (flipped) phase. Numerical parameters: spectral correlation Δ *v _c_* = 250 *GHz*, number of generations 10000, size of population per generation 100.

As an initial test case, we targeted the ability to simultaneously focus the transmitted field in space and time (spatiotemporal focusing). Our simulation results are shown in Figure 2. As an input field, we considered a single-cycle, transform-limited THz pulse ( Figure 2a). As illustrated in Figure 2b and c, the corresponding transmitted wavefront for a non-optimised pattern is generally spread in time ( Figure 2b) and scattered in space ( Figure 2c). To obtain a spatiotemporal focus, we applied our GA algorithm to maximise the cost function A, where max *E*_0_( *t*) ≡ max *E*( *x*_0_, *y*_0_, *t*) is the peak electric-field value in the desired focal spot ( *x*_0_, *y*_0_). Such a fitness function simultaneously maximises the transmitted field's peak value and minimises the pulse profile's standard deviation in time.

A typical optimised pulse is shown in Figure 2e and f. showcasing our ability to simultaneously focus and recompress the transmitted pulse in the desired focal spot. The ability to restore the spectral properties of the pulse is particularly evident by comparing the spectrum of the optimised pulsed ( Figure 2d, solid green line) with the incident one ( Figure 2e, blue dashed line). Quantitatively, the optimised spectrum has an FWHM band of 0.52 *THz* (input 0.8 *THz*). Quite interestingly, our approach is sensitive to the coherent field properties of the pulse, as illustrated in Figure 2g and h, where we demonstrate the ability to flip, or invert, the optical phase of the THz pulse by employing the fitness function B from Table 1. The corresponding spectral profile is shown in Figure 2d (orange line).

**Figure 3.**
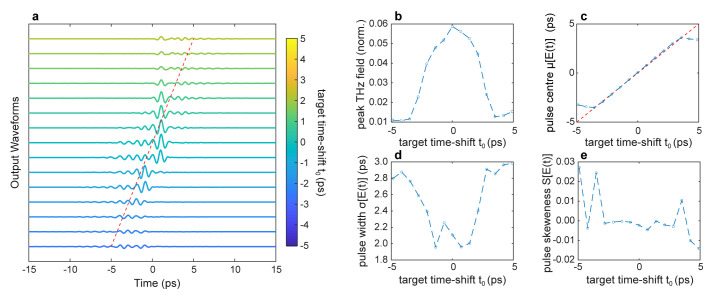
Time shift control of a THz pulse. **a**. Temporal profiles of the output THz pulses optimised for different values of time delay *t*_0_. **b**. THz peak field as a function of the time delay *t*_0_. **c** First central moment *μ*, corresponding to the pulse centre, as a function of the time delay *t*_0_. The red dashed line represents the desired time-delay *μ* = *t*_0_. **d**. The second central moment *σ*, corresponding to the pulse width, as a function of the time delay *t*_0_. **e** Third central moment *S*, corresponding to the pulse symmetry, as a function of the time delay *t*_0_. Numerical parameters: spectral correlation Δ *v _c_* = 250 *GHz*, number of generations 20000, size of population per generation 50, and weight factors { *α*, *β*, *γ*, *ζ*} ≡ {10 ^2^, 5 ⋅ 10 ^12^, 10 ^12^, 1}.

Figure 3 illustrates the ability to simultaneously optimise several temporal properties of the transmitted wavefront in a single GA optimisation cycle. For this scenario, we employed the multi-objective optimisation fitness function C in Table 1, which maximises the peak field in time while simultaneously searching for incident patterns yielding an output wavefront with the desired centre *t*_0_, duration *σ*_0_ and skewness *S*_0_. Specifically, we performed a series of simulations with a target duration *t*_0_ and skewness *S*_0_ coinciding with the transform-limited incident pulse, while we varied the target time-shift within the interval *t*_0_ ∈ [–5 *ps*, 5 *ps*]. The factors { *α*, *β*, *γ*, *ζ*} multiplying each term are the cost function’s weights. As illustrated in Figure 3a, the pulse can be simultaneously recompressed and controllably time-shifted in a similar fashion to the case studied in Ref. 25.

However, the ability to control the pulse centre comes at the price of a reduced ability to achieve a good degree of spatiotemporal focusing, as can be evinced by assessing the properties of the optimised wavefronts ( Figure 3b–e). While in our simulations, we achieved a broad degree of control of the centre of the pulse *μ* ( Figure 3b), we observed an overall decrease in the peak field ( Figure 3b) and an increase in the pulse duration ( Figure 3d). This behaviour is in good qualitative agreement with the experimental results of Ref. 25. However, in the framework of THz-TDS, these results suggest that one could ideally scan the THz pulse profile within a range of a few ps without mechanical time-delay devices (i.e., translation stages), commonly used in ultrafast optical setups.

### Field-sensitive spectral shaping: chirp and CEO control

A key advantage of our methodology is the possibility of detecting the coherent properties of the transmitted THz pulse. These correspond, in particular, to the spectral amplitude and absolute phase, which are generally not directly measurable quantities in experiments at optical frequencies.

The ability to coherently resolve the complex-valued spectrum of the transmitted waveforms is particularly suited to perform phase-sensitive spectral shaping of the incident pulse. For this type of task, we employ the cost function D in Table 1, where *E _target_* ( *ω*) is the desired spectral profile, *E _out_* ( *ω*) is the output spectrum, and σ( *E*( *t*)) denotes the standard deviation of the pulse in the desired focal point.

**Figure 4.**
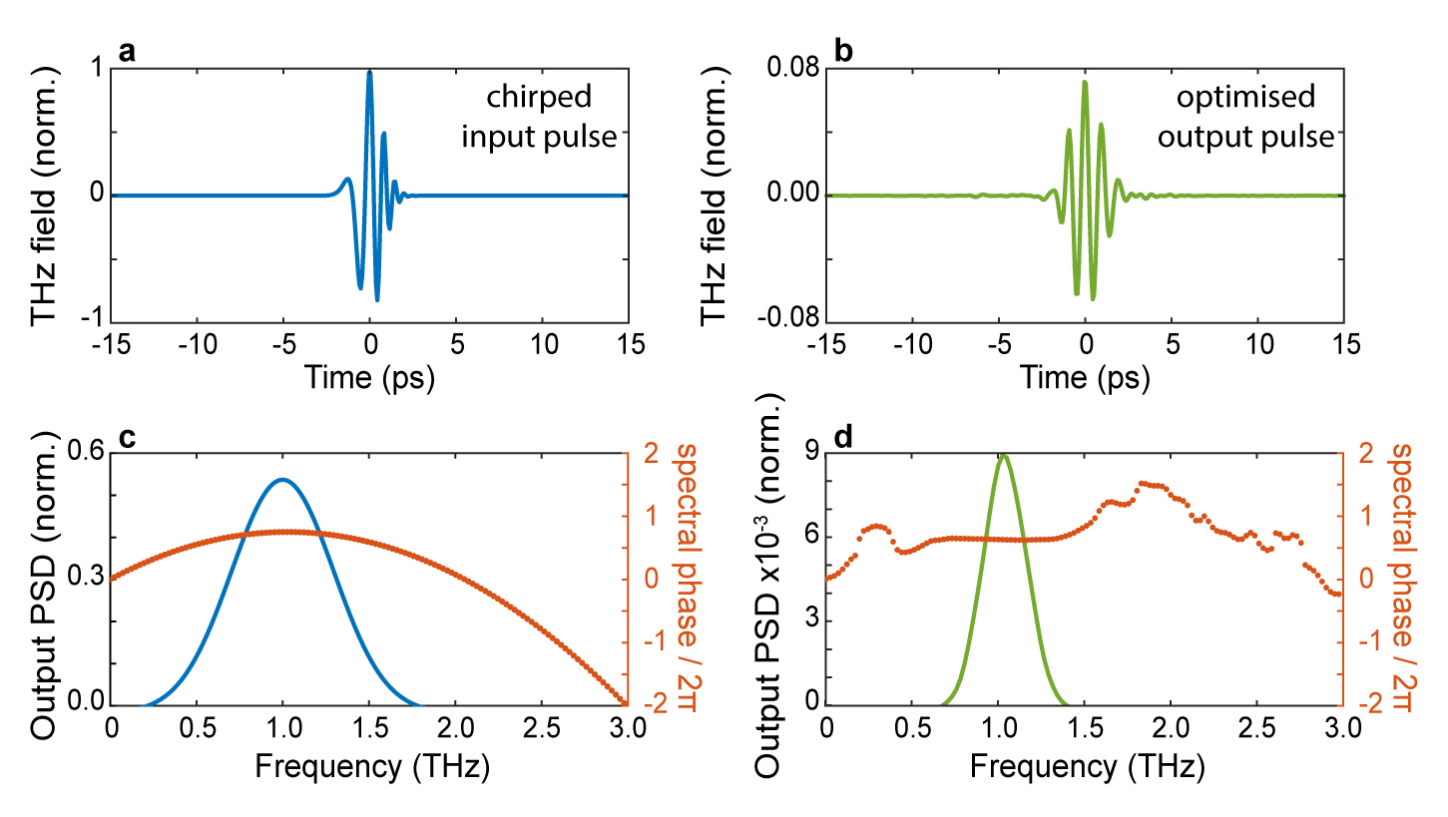
Chirped-pulse compression. **a**. The input temporal trace of an initially chirped pulse. **b**. Temporal trace of the optimised pulse. **c**. Power spectral density of the input pulse (blue line) and spectral phase (orange dots). **d**. Power spectral density of the optimised pulse (blue line) and spectral phase (orange dots). The algorithm tries to fit the output spectral phase as close as possible to the phase of a transform-limited pulse. Numerical parameters: spectral correlation Δ *v _c_* = 250 *GHz*, number of generations 10000, size of population per generation 300.

**Figure 5.**
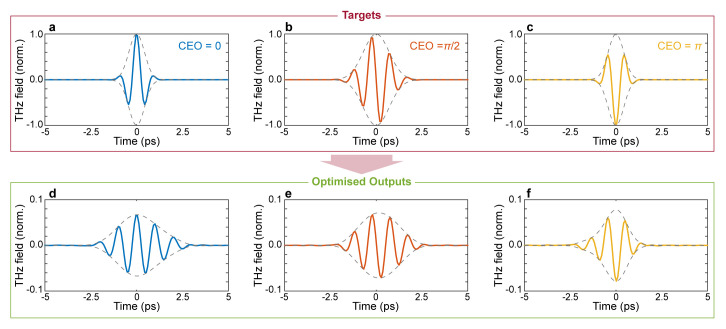
Carrier envelope offset (CEO) modulation. Temporal profiles of target and optimised pulses for CEO phases *ϕ* = 0. ( **a**, **d**), $\phi =\frac{\pi }{2}$ ( **b**, **e**), and *ϕ* = *π* ( **c**, **f**). For all cases, the input field is the transform limited pulse shown in Figure 2a. Numerical parameters: spectral correlation Δ *v _c_* = 250 *GHz*, number of generations 50000, size of population per generation 50.

This particular choice of fitness function selects patterns yielding output waveforms with a short duration and a high degree of spectral correlation with the desired waveform. As a first example, in Figure 4, we illustrate the possibility of ‘recompressing’ at the output of the scatterer an incident pulse characterised by a linear chirp. The field profile of the incident pulse is shown in Figure 4a, while the spectral amplitude (blue line) and absolute phase (orange dots) are shown in Figure 4c, respectively. In this scenario, we choose the transform-limited version of the incident pulse as the target waveform in Figure 4a. As can be readily evinced from the optimised pulse profile shown in Figure 4b, in our simulations, we were able to restore the symmetry of the optimised wavefront, albeit for a slightly longer pulse. The ability to flatten the spectral phase is clearly shown in Figure 3d, where we report the spectral amplitude and absolute phase of the optimised pulse. Interestingly, our approach to pulse shaping can be directly applied to achieve extremely fine control of the absolute phase of the output waveform. As a final example, we assessed the possibility of finely tuning the CEO of the optimised waveform. Our results are shown in Figure. 5. In all four cases, we considered an input field corresponding to a transform-limited THz pulse (identical to the case in Figure 2a). As output wavefront targets, we considered waveforms with different CEO values ( Figure 5a–c). The corresponding optimised output fields, illustrated in Figure 5d–f, demonstrate the ability to finely tune the CEO of the output waveform, showcasing a remarkable ability to manipulate the coherent properties of the incident pulse.

## Discussion and conclusions

In this work, we theoretically investigate a field-sensitive wavefront shaping methodology to control the spatiotemporal properties of terahertz pulses transmitted through a scattering medium. Our approach combines the field detection capabilities of TDS techniques with the generation of terahertz structured beams through nonlinear conversion of optical beams. Our simulations show how the full access to the coherent properties of the transmitted terahertz field enables defining phase-sensitive evolutionary optimisation strategies to manipulate the carrier-wave properties of THz pulses as they travel through complex media. As in relevant case studies, we considered different wavefront control applications, including spatiotemporal focusing, phase inversion, temporal shifting control, and re-compression of a chirped THz pulse. Quite remarkably, we demonstrated the ability to manipulate the CEO of a single-cycle incident pulse. Controlling the CEO of ultrafast pulses is a highly challenging task in photonics that usually requires advanced detection techniques such as *f-2f* interferometry ^60– 62^. Our results differ from traditional spatiotemporal focusing at optical and infrared frequencies as THz-TDS detection provides a direct, coherent measure of the properties of the transmitted electric field. Such a remarkable result would have a profound impact, especially for THz imaging, since time-resolved characterisation techniques are currently highly desired due to the broad spectrum of potential applications, including deep-tissue biological imaging and time-reversal control of optical waves ^63, 64^. Although we did not provide specific modelling for near-field interactions in this work, the access to the near field modes is less challenging at terahertz frequencies. Hence, it is to be noted that if the THz source is located in near-field conditions with the sample and spatially controlled with NGI-methodology, the accessible modes within the scattering medium can potentially focus light under the diffraction limit, a potential route to subwavelength imaging resolution. Moreover, TDS-based wavefront control techniques could provide a practical approach to manipulating broadband terahertz pulses' spatiotemporal properties and design entirely new classes of wavefront and spectral shaping applications. It is also important to highlight that due to the scale of the wavelengths, working at THz frequencies offers a considerable advantage to have a precise and determinist approach in sample realisation (e.g., 3D printing) ^65, 66^. Additionally, the size of each scattering particle would be bigger (i.e., hundreds of micrometre ^45^) compared to what is usually used in optical frequencies (e.g., nanometre-scale ^67^), therefore rendering samples intrinsically more robust to thermal and mechanical effects. Finally, the combination of THz technology with concepts and methodologies from complex photonics opens up the intriguing (and unexplored) possibility of performing time-resolved, full-wave scattering matrix retrieval experiments.

## Data availability

### Underlying data

Figshare: Figure data for "Nonlinear field-control of terahertz waves in random media for spatiotemporal focusing". http://doi.org/10.6084/m9.figshare.19096859 ^57^.

This project contains the following underlying data, organised by folder:

-    Figure 2/

○ mat/ (folder containing raw datasets (in .fig and .mat formats) for each panel in Figure 2)

-    Figure 3/

○ mat/ (folder containing raw datasets (in .mat format) for each panel in Figure 3)

-    Figure 4/

○ mat/ (folder containing raw datasets (in .fig and .mat formats) for each panel in Figure 4)

-    Figure 5/

○ mat/ (folder containing raw datasets (in .fig and .mat formats) for each panel in Figure 5)

### Extended data

Figshare: Figure data for "Nonlinear field-control of terahertz waves in random media for spatiotemporal focusing". http://doi.org/10.6084/m9.figshare.19096859 ^57^.

This project contains the following extended data, organised by folder:

-    Figure 2/

○ make_f2.m (MATLAB script that reads and processes the raw data and plots the figure)
○ make_f2_octave.m (Octave script that reads and processes the raw data and plots the figure)

-    Figure 3/

○ make_f3.m (MATLAB script that reads and processes the raw data and plots the figure)
○ make_f3_octave.m (Octave script that reads and processes the raw data and plots the figure)

-    Figure 4/

○ make_f4.m (MATLAB script that reads and processes the raw data and plots the figure)
○ make_f4_octave.m (Octave script that reads and processes the raw data and plots the figure)

-    Figure 5/

○ make_f5.m (MATLAB script that reads and processes the raw data and plots the figure)
○ make_f5_octave.m (Octave script that reads and processes the raw data and plots the figure)

-    simulation_codes_octave/

○ Main_costfunction_A.m (simulation code with cost function A from Table 1)
○ Main_costfunction_B.m (simulation code with cost function B from Table 1)
○ Main_costfunction_C.m (simulation code with cost function C from Table 1)
○ Main_costfunction_D.m (simulation code with cost function D from Table 1)
○ rsgeng1D.m (function to generate gaussian-correlated 1D data)
○ TM_f.m (function to generate a frequency-correlated transmission matrix)
○ fwhm.m (function to estimate the FWHM of a temporal pulse profile)
○ compute_moment.m (function to calculate the statistical moments of temporal pulse profiles)
○ spectr.m (function to calculate the Fourier spectrum of a temporal field profile)
○ erdc_fireice_h.m (utility function for customised colormap)
○ fireice.m (utility function for customised colormap)

Data and simulation codes are available under the terms of the Creative Commons Attribution 4.0 International license (CC-BY 4.0).

## Ethics and consent

Ethical approval and consent were not required. 
