# Supplementary material for: Nonlinear field-control of terahertz waves in random media for spatiotemporal focusing
Source: Open Res Eur. 2023 Feb 13;2:32. Originally published 2022 Mar 8. [Version 3] doi: 10.12688/openreseurope.14508.3 (PMC10445851; doi:10.12688/openreseurope.14508.3)
Supplement: Peer review history

# Peer review: Reviewer response for version 2

**DOI:** 10.21956/openreseurope.16219.r30397

**Published:** 2022-12-14

**Authors:** Silvia Gentilini

**Affiliations:** 1 Institute for Complex Systems, National Research Council (ISC-CNR), Rome, Italy

The manuscript "Nonlinear field-control of terahertz waves in random media for spatiotemporal focusing" presents a theoretical and numerical demonstration of a technique for the full-wave control of a Terahertz pulse propagating in a random media. In the last ten years, the spatial manipulation of an electromagnetic wave to control its propagation through turbid media has raised many researchers' interest in photonics. This has brought significant improvement in the techniques aimed at overcoming and eventually exploiting the structural disorder at the scale of optical wavelength. At these frequencies, the success of these techniques is mainly due to the availability of spatial light modulators (SLM) that, combined with optimizing algorithms, enabled an approach to the inverse scattering problem and obtained results such as super focusing and imaging through opaque media. In this framework, the novelty of the work authored by Cecconi et al. is to achieve spatio-temporal shaping of ultra-short pulse in the THz range, combining the well-established outcomes obtained to control the spatial distribution of optical field with the advantage of the full-field detection of time-domain-spectroscopy (TDS). Specifically, the Authors propose using an optical SLM to modulate the wavefront of the optical pump that, impinging on the nonlinear crystal that generates the THz pulse, transmits to this latter the modulation. The optimization of such a modulation is obtained by a scheme that foresees feedback, provided by a genetic algorithm, between the pulse transmitted and the pattern to impress on the optical pump . 

 The Authors simulated some test cases to demonstrate the validity of their theoretical approach and of the proposed scheme to realize it.

 Specifically by playing with the genetic algorithm (GA) chosen as optimization algorithm, they demonstrate e sub-wavelength focusing of the pulse and simultaneously the ability to control the pulse centre, keeping fix the focusing point. Remarkable is also that, leveraging the phase sensitive TDS detection and by suitable choice of the fitness function of the GA, they succeed in optimizing the spectral shape of the pulse transmitted by the disordered sample performing a control on the chirp of the pulse and of its carrier-envelope-phase.

 The work is reported with accuracy and provides details helpful to interested researchers to implement the clever strategy proposed for other applications in the THz range. 

 The work also raised a series of interesting question to address in future as the existence of a disorder threshold to use the methodology. I'm also wondering if it was possible to implement the technique to obtain multi-point focusing by translating the collection system in order to obtain a scan of the output surface.

 Below I report some minor revisions that the Authors could address in the text:

1. I don't understand the meaning of the infinity symbol in Eq.(4).
2. Some misreferences to the figures in the text. 
  - In the second paragraph of the Results, on page 5, the Authors refer to Figure 2e for the spectrum of the incident beam. I think that the correct reference figure is Figure 2d.
  - In the second paragraph on page 7, the Authors refer to Figure 3b for the pulse centre. I think that the correct reference is Figure 3c.
3. In the title of the second subsection of the "Results", I think the CEO has to be changed in CEP.

Is the study design appropriate and does the work have academic merit?

Yes

Is the work clearly and accurately presented and does it cite the current literature?

Yes

If applicable, is the statistical analysis and its interpretation appropriate?

Yes

Are all the source data underlying the results available to ensure full reproducibility?

Yes

Are the conclusions drawn adequately supported by the results?

Yes

Are sufficient details of methods and analysis provided to allow replication by others?

Yes

Reviewer Expertise:

Photonic of complex systems

I confirm that I have read this submission and believe that I have an appropriate level of expertise to confirm that it is of an acceptable scientific standard.

# Response

**Published:** 2023-01-27

**Authors:** Juan Sebastian TOTERO GONGORA

**Affiliations:** Loughborough University, UK

We are glad that the reviewer appreciated the work we have performed, and we thank the reviewer for their positive assessment. In relation to the interesting suggestion to obtain multi-point focusing (e.g., to perform a raster scan of a sample), in our approach, it is possible to focus the THz pulse in different spatial target points by writing different cost functions related to different spatial positions. The ability to manipulate different points of the scattered field is indeed a key advantage of using an iterative optimisation algorithm, and it could enable us to find the optimal patterns producing foci in different points. We note, however, that in actual experiments finding an optimal solution far away from the centre of the optical path might be more challenging. In the presence of non-uniform illumination, for example, points close to the edge of the illumination region might be characterised by a lower signal-to-noise ratio. To clarify this point, we added the sentence “While our analysis was conducted by considering a target focal point placed in the centre of the optical path, the use of an iterative optimisation approach enables, in principle, to select different points on the output plan. In practical terms, this would correspond to modifying the cost functions in Table 1 to consider the scattered electric field at different positions. We note, however, that in real-life experiments, the ability to obtain an optimal solution at any point on the output plane might be restricted by the properties of the scattering medium and the uniformity of the optical illumination.” in the Discussion and Conclusions section. Please find below our point-by-point answer to the further suggestion for revision:

 1) Regarding the infinity symbol in equation (4), it is a rendering error when generating the PDF. We thank the reviewer for pointing it out and have amended it in the new version.

 2, 3) Thanks for the note; we have corrected the references.

 4) Regarding the title of the second subsection of the ‘Results’, the Referee is correct. We changed it to ‘Field-sensitive spectral shaping: chirp and CEP control’.

# Peer review: Reviewer response for version 2

**DOI:** 10.21956/openreseurope.16219.r29901

**Published:** 2022-08-31

**Authors:** Romain Peretti

**Affiliations:** 1 IEMN - CNRS, Villeneuve d’Ascq, France

The manuscript "Nonlinear field-control of terahertz waves in random media for spatiotemporal focusing" describes a theoretical and numerical work describing an innovative implementation of super focusing in scattering disordered media in the terahertz frequency band. Imaging and tight focusing directly inside disordered media was assumed to be impossible mostly due to the multiple scattering events making the photon path almost impossible to predict. However since the scattering processes remain coherent and time reversible, works of the past decades showed that thanks to an inverse problem approach one can retrieve information at a very high resolution. Such an approach had several theoretical outcomes and applied consequences as in Optical coherence tomography. One of the key levers to achieve such an approach is to control the shape of the wave front of the beam. The author of the papers propose a very smart way to do it using nonlinear generation of Terahertz radiation and instead of imprinting the pattern on the THZ beam they proposed to imprint it on the infrared beam used to generate the THz through nonlinear optics effect. In this way, one only needs conventional spatial light modulators and not THz ones.

 The manuscript provides numerical analysis based on simulated scattering samples showing subwavelength focusing on a broad band. Not only that but the authors managed to focus, but they also shifted the pulse to a desired time delay in a few picoseconds range and modulated the carrier envelope phase. The conclusions are well supported and the text is very clear.

 This work is a real advance in the field and would suggest many questions among them :

 If scattering is more and more understood in the visible and near infrared with scatterers corresponding to this wavelength range, it is less the case for the scatterer in the THz frequency ranges. The model proposed are well known in the higher energy band but I wonder if they have already been applied in the THz since ref 37 to 40 of the papers does not discuss it. In other words, are there any arguments for this model to work in the THz range especially compared to the analysis in ref 37?

 Is it possible to focus the THz spot elsewhere than in the middle of the optical path?

Is the study design appropriate and does the work have academic merit?

Yes

Is the work clearly and accurately presented and does it cite the current literature?

Yes

If applicable, is the statistical analysis and its interpretation appropriate?

Not applicable

Are all the source data underlying the results available to ensure full reproducibility?

Yes

Are the conclusions drawn adequately supported by the results?

Yes

Are sufficient details of methods and analysis provided to allow replication by others?

Yes

Reviewer Expertise:

Terahertz Photonics

I confirm that I have read this submission and believe that I have an appropriate level of expertise to confirm that it is of an acceptable scientific standard.

# Response

**Published:** 2023-01-27

**Authors:** Juan Sebastian TOTERO GONGORA

**Affiliations:** Loughborough University, UK

We thank the reviewer for the thorough analysis of our manuscript and their positive assessment of our results. In the following, we address the reviewer’s comments:

 1) Thanks for the interesting question. As correctly pointed out by the reviewer, there are no experimental studies on the broadband transmission matrix representation of a scattering medium at THz frequencies. In the state-of-the-art (cf. Refs. 37-40), researchers have mostly analysed the scattering response in terms of average diffusion models by collecting the total transmitted field at the output. A key challenge in obtaining a full transfer matrix representation of the scattering is the requirement of resolving the scattered electric field in space, which is extremely challenging in THz experiments. At optical frequencies, however, it has been shown that the transfer matrix representation is equivalent to the diffusion approximation results for a space-dependent response. The transfer matrix framework,  in general, is derived and validated in accordance with the diffusion approximation [R1]. The experimental validation of our transfer matrix model will be the subject of our future work. Our objective in this manuscript is to review the theoretical implications of time-resolved, field-sensitive detection for manipulating broadband THz pulses, and until now, no complete, field-based wavefront control methodology has been developed for manipulating broadband pulses at THz frequencies.

 2) We thank the author for raising this excellent point. In our approach, it is possible to focus the THz pulse in different spatial target points by writing an appropriate cost function. The ability to manipulate different points of the scattered field is indeed a key advantage of using an iterative optimisation algorithm. In practical terms, this would correspond to changing the desired focal point where E0 is calculated or expressing different cost functions for different points at the output. We note, however, that in actual experiments finding an optimal solution far away from the centre of the optical path might be more challenging. In the presence of non-uniform illumination, for example, points close to the edge of the illumination region might be characterised by a lower signal-to-noise ratio. To clarify this point, we added the sentence “While our analysis was conducted by considering a target focal point placed in the centre of the optical path, the use of an iterative optimisation approach enables, in principle, to select different points on the output plan. In practical terms, this would correspond to modifying the cost functions in Table 1 to consider the scattered electric field at different positions. We note, however, that in real-life experiments, the ability to obtain an optimal solution at any point on the output plane might be restricted by the properties of the scattering medium and the uniformity of the optical illumination.” in the Discussion and Conclusions section.   [R1] S M Popoff, et al., 2011 New J. Phys. 13 123021. DOI 10.1088/1367-2630/13/12/123021
